# Low Threshold Room Temperature Amplified Spontaneous Emission in 0D, 1D and 2D Quantum Confined Systems

**DOI:** 10.1038/s41598-018-22287-9

**Published:** 2018-03-02

**Authors:** Parva Chhantyal, Suraj Naskar, Tobias Birr, Tim Fischer, Franziska Lübkemann, Boris N. Chichkov, Dirk Dorfs, Nadja C. Bigall, Carsten Reinhardt

**Affiliations:** 1 Laser Zentrum Hannover e.V., Nanotechnology Department, Hannover, D-30419 Germany; 2 Leibniz Universität Hannover, Institute of Physical Chemistry and Electrochemistry, Hannover, D-30167 Germany; 3 Laboratory for Nano and Quantum Engineering, Hannover, D-30167 Germany; 4 University of Applied Sciences, Bremen, D-28199 Germany

## Abstract

We address optical amplification properties of quantum nanoparticles of the cadmium selenide/cadmium sulfide (CdSe/CdS) material system with different dimensionality of spatial confinement. CdSe/CdS core/shell quantum dots (QDs), core/shell quantum rods (QRs) and 5 monolayer thick core/crown nanoplatelets (NPLs) at ambient temperature are considered, exhibiting 0D, 1D and 2D spatial confinement dimensionality of the electronic system, respectively. Continuous films of all these nanoparticles are synthesised, and amplified spontaneous emission (ASE) spectra are measured under femtosecond pumping at wavelengths of 400 nm and 800 nm, respectively. The lowest threshold is found for NPLs and the highest for QDs, demonstrating the influence of the rod-like and plate-like CdS structures. To emphasize this effect, ASE is demonstrated also in CdSe/CdS QRs and NPLs under nanosecond pumping at 355 nm in the same material films. The amplification has been achieved without use of any feedback structure, emphazising the efficiency of the antenna effect. The pumping threshold fluences for NPLs and QRs are observed to be similar, but no ASE is observed in QDs up to the damage threshold of the nanoparticle layers. The length variation investigation with nanosecond pumping resulted in the gain coefficients of 29 *cm*^−1^ and 37 *cm*^−1^ for QRs and NPLs, respectively.

## Introduction

Colloidal semiconductor nanoparticles have become a topic of huge interest in recent years due to their quantum confinement and optical properties, making them interesting candidates for the realization of novel active photonic components and devices^1,2^. As the size of the nanoparticles decreases, discrete energy levels in the electronic system appear, which provide possibilities for fluorescent emission and light amplification in the visible spectral regime^3–9^. According to the Brus equation^10^, the energy band gap of small spherical quantum dots (QDs) is proportional to 1/*R*^2^, where, *R* is the radius of the particle. The emission wavelength can thus be influenced by the degree of confinement, providing huge potential for the development of novel integrated photonic light sources. This property endorses huge advantage in chip-scale photonic data transport and processing, telecommunication as well as medical applications^3^.

However, when the size of the particles decreases the quantum confined materials become more susceptible to strong size- and temperature-dependent Auger recombination (AR)^11–13^. This process interrupts normal exciton mobility by imposing extra losses. The Coulomb interaction between the electron-hole pairs (excitons) is interrupted by the AR, resulting in increased heating of the material^3,14,15^. A previous research done on cadmium selenide/zinc sulfide (CdSe/ZnS) quantum rods (QRs) and QDs revealed that AR in QDs occurs on the picosecond timescale, which is faster than the exciton radiative lifetime in the order of nanoseconds^5^. This enforces fast reduction of the optical gain and, as a result, only fluorescence from the materials can occur after optical pumping using long pulse durations in the order of nanoseconds. Nevertheless, amplified spontaneous emission (ASE) can still be achieved if cumulative stimulated processes among many QDs occur faster than the Auger recombination. Since the rate of the stimulated emission buildup is proportional to the QDs concentration in the sample^3^, it could be demonstrated that by close packaging of QDs into solid state films, it is possible to obtain the concentrations that are sufficiently high for the optical gain to successfully compete with the Auger decay^16^. Besides, the use of solid films is beneficial to obtain optical gain when the pumping process is faster than the AR at room temperature or when the material is cryogenically cooled. One of the common ways to achieve optical amplification in such quantum materials is by using high power excitation pulses with pulse durations of a few tens or hundreds of femtoseconds^17^. While the use of highly expensive femtosecond laser systems is still appropriate for basic studies of the optical properties and their ability to provide optical gain, it would be desirable for practical applications to control and pump optical devices on the basis of quantum materials with low-cost nanosecond pump sources. Recently low-threshold lasing in CdSe/ZnS QDs embedded into a thin layer of PMMA has been demonstrated under nanosecond pumping at 355 nm using a distributed feedback structure in order to benefit from low gain^18^. Similarly, amplified emission from QDs was measured with 500 nm (9 ns) laser using a distributed Bragg reflector to enhance modal gain^19^.

In order to reach higher optical gain in quantum confined material systems, nanoparticles of different confinement dimensionality can be considered. The rate of AR strongly depends on this confinement dimensionality and is expected to be significantly slower in 1D QRs and 2D nanoplatelets (NPLs), respectively, compared to 0D QDs^5^. This effect of the carrier confinement dimensionality on AR by tuning the confinement regime has been demonstrated in the CdSe material system considering 0D QDs and 1D QRs^13^. It has been found that QRs show lower AR and thus higher amplification at the same emission wavelengths, which can be explained by a significant shortening of the exciton radiative lifetime^20^ and a reduced Auger heating rate^21^. In addition to the reduced AR influence, the 1D QRs and 2D NPLs show an enhanced absorption cross section compared to 0D QDs, where the rod and shell structures are acting as broadband optical antenna^5^ along with a higher carrier concentration, contributing to the excitons, providing high optical gain^3^. Small NPLs, due to their 2D spatial extension, have been demonstrated to exhibit an even larger absorption cross-section as compared to QRs and QDs^22^. This enables achieving higher gain and ASE without any resonator structure using lower pump power laser sources.

The comparison of the influence of the dimension of confinement for one material system is however of utmost importance. Since the overall ASE performance of a given nanoparticle film strongly depends on various often only poorly known parameters such as packing density, optical homogeneity and many more, a comparison of a given particle geometry with data from other groups (from the literature) for a different particle geometry will always be misleading since the mentioned parameters are often not comparable. To our knowledge, a direct comparison of optical amplification properties in semiconductor quantum nanoparticles of different particles dimensionality, excited by different pump laser sources in a same material system is not demonstrated so far. In this report, we demonstrate ASE in the cadmium selenide/cadmium sulfide (CdSe/CdS) material system with different dimensionality. Optical gain has been achived in high-quality spin-coated films of CdSe/CdS core/shell QDs, core/shell QRs and core/crown 5 monolayer NPLs und femtosecond pumping using one- and two photon absorption. Furthermore, optical gain is demonstrated in the same QRs and NPLs material films under nanosecond pumping without the use of any feedback structure, paving the way for practical applications of quantum confined materials as versatile light sources.

All films were produced by identical procedures and measured under identical conditions, giving rise to a robust analysis of the influence of the particle geometry on the ASE performance. The optical emission properties of three quantum nanoparticles of the CdSe/CdS material system with different dimensionality of spatial confinement are measured. The CdSe/CdS core/shell QDs, core/shell QRs and core/crown 5 monolayer NPLs at ambient temperature exhibit 0D, 1D and 2D spatial confinement dimensionality of the electronic system, respectively. Continuous films of these nanoparticles are synthesized, and ASE spectra are measured under femtosecond pumping at wavelengths of 400 nm and 800 nm, respectively, as well as nanosecond pumping in the ultraviolet spectral range at 355 nm. The threshold power to achieve ASE for each nanoparticle are characterized and compared. ASE in CdSe/CdS nanoparticles of all three confinement types is confirmed for pumping at 400 nm and 800 nm, using 50 *fs* pump pulses. For a pump wavelength of 400 nm the absorption corresponds to a single-photon process, giving the lowest threshold values of ≈542 *μ*J*cm*^−2^, ≈25 *μ*J*cm*^−2^ and ≈15.42 *μ*J*cm*^−2^ for QDs, QRs and NPLs, respectively. These values correspond to a length of the pumped zone of 1.5 mm. A pump wavelength of 800 nm requires a two-photon absorption process, which provides ASE thresholds of ≈35 mJ*cm*^−2^, ≈20 mJ*cm*^−2^ and ≈10.5 mJ*cm*^−2^ for QDs, QRs and NPLs, respectively. The lowest threshold found for NPLs indicates the positive influence of the rod-like and plate-like CdS structures. In order to demonstrate the huge potential of this material system ASE is demonstrated in homogeneous films of CdSe/CdS QRs and NPLs under nanosecond pumping at 355 nm at ambient temperature. The optical gain is found to be high enough that no resonator structure is necessary for achieving amplification of spontaneous emitted photons. ASE pump power thresholds for the QRs and NPLs are ≈1.3 mJ*cm*^−2^ and ≈1.54 mJ*cm*^−2^, respectively. No ASE is observed in QDs up to the damage threshold of the nanoparticle layers around ≈22 mJ*cm*^−2^.

## Results and Discussion

The objective of the experimental investigations presented in this contribution is to study the influence of the confinement dimensionality on the light emission properties of nanoparticles of a single material system under femtosecond and nanosecond optical pumping. The nanoparticles under consideration consist of CdSe/CdS core/shell QDs, core/shell QRs and core/crown 5 monolayer NPLs. All nanoparticle samples provide a high absorption cross section in the UV spectral range for efficient one-photon absorption pumping. Films of the materials were spin-coated to layer thicknesses between 150 nm and 190 nm onto 150 *μ*m thick glass substrates. For optical pumping, the samples were illuminated by the second harmonic and the fundamental wave of a mode-locked amplified titanium:sapphire laser (Spitfire), operating at a central wavelength of 800 nm with a pulse duration of 50 fs and repetition rates of 20 Hz and 50 Hz, respectively, as well as the third harmonic of a nanosecond Nd:YAG laser (Innolas Spitlight DPSS) with 6 ns pulse duration and 14 Hz repetition rate.

**Figure 1:**
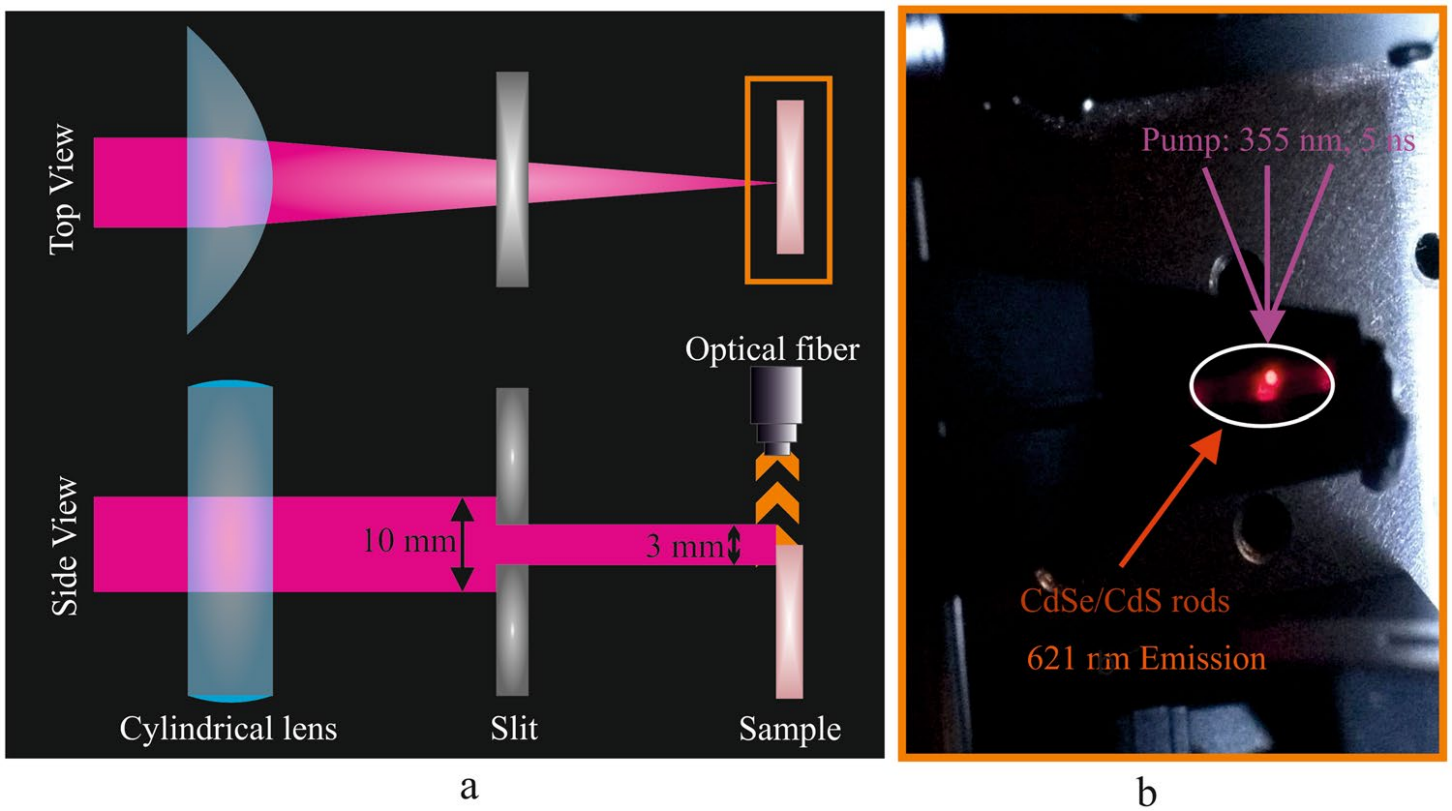
(**a**) Schematic diagram of the experimental set up, containing a laser beam focused by a cylindrical lens from top view and side view (with a metal slit fully opened) to control the focus length on a sample. The photoluminescence is measured by positioning an optical fiber perpendicularly, close to the edge of the film. (**b**) Experimental photo of CdSe/CdS rods, excited with 355 nm (6 ns) laser. The photo shows a view onto the upper edge of the sample. The bright spot inside the white circle is the ASE from the pumped zone, demonstrating the strong emission and amplification properties.

For investigations, the laser radiation was focused to a line onto the sample surface by a cylindrical lens with a focal length of 5 cm. The length of the illuminated line was varied by means of a mechanical slit inserted after the cylindrical focusing lens, as shown in Fig. 1(a). The beam diameters in front of the lens were 10 mm with approximately constant intensity. The diameter of the focal line was determined to 45 *μ*m, and the length of the focal line was varied by a micrometer-driven metal slit. The middle of the focal line was positioned onto an edge of the material samples, so that the optically pumped line extends into the material layer when the slit was opened. The maximum opening of the slit was 3 mm, resulting in a maximum interaction length on the sample of up to 1.5 mm. The laser power was varied by means of dielectric optical attenuators. The emission spectra of the nanoparticle films were measured by positioning the fiber of a spectrometer (Ocean Optics) with a core diameter of 200 *μ*m above the edge of the pumped zone of the sample. The spectrometer has an accuracy of ±2 nm, which is the error in the wavelength measurements given below. The emission from the upper edge of the pumped zone was clearly visible with the naked eye, as shown in Fig. 1(b).

First experiments were performed using the second-harmonic output of the titanium:sapphire laser at 400 nm, providing one-photon absorption pumping of the individual nanoparticle samples. Using the maximum interaction length of 1.5 mm, the pulse energy of the second-harmonic (2*ω*) output was increased until a definite evidence of a sharp and narrow peak was observed on top of the spectrally broad fluorescence emission. The spectral narrowing during the transition from the fluorescence to the amplification regime clearly indicates the onset of ASE^23^.

**Figure 2:**
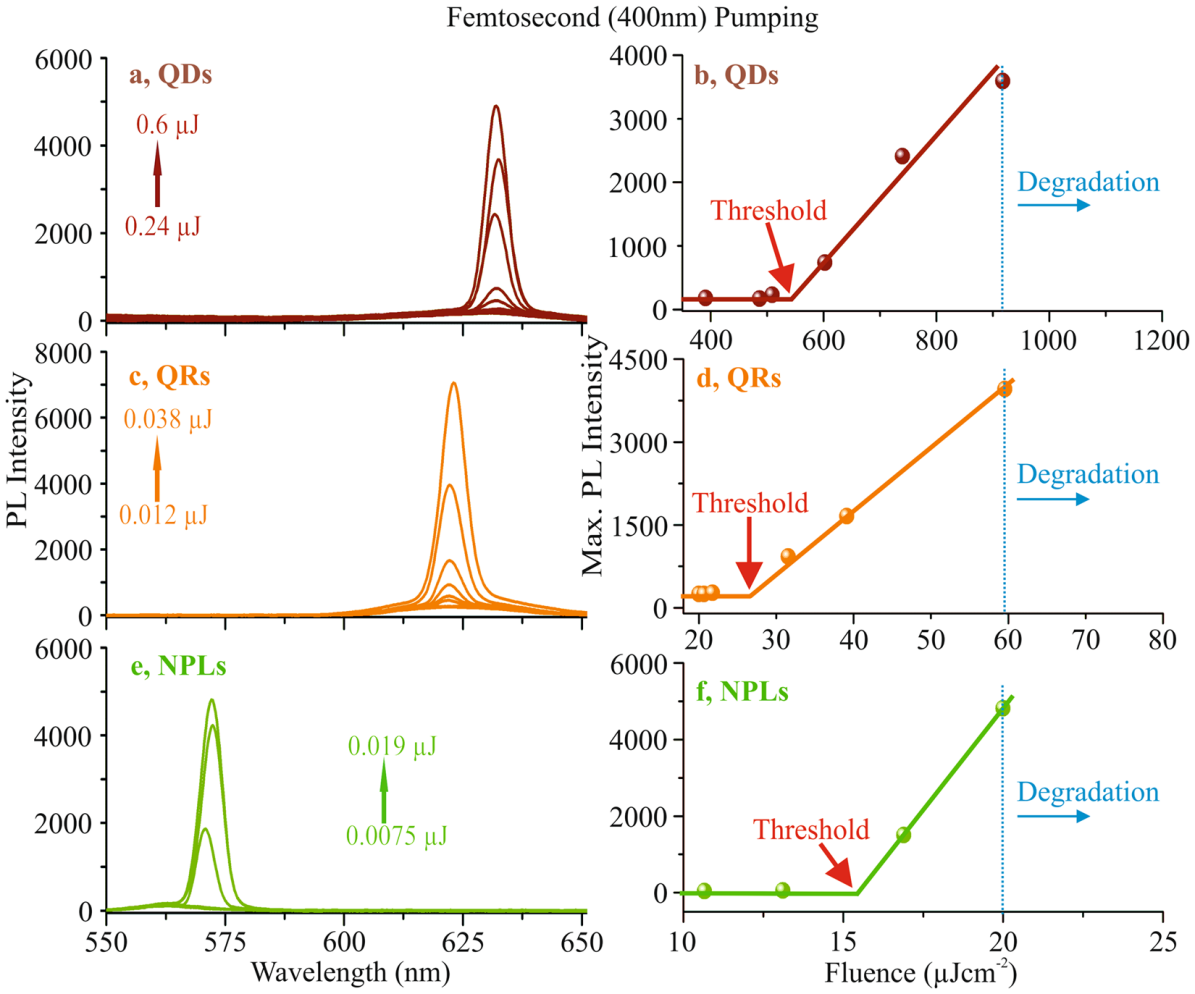
The comparison between QDs (**a**,**b**), QRs (**c**,**d**) and NPLs (**e**,**f**), measured with 2*ω* pumping. The arrows in each graph (red for QDs, orange for QRs and green for NPLs) represent the energy range used. All three nanoparticles show clear indication of ASE at the peak wavelengths of 632 nm (QDs), 623 nm (QRs) and 572 nm (NPLs). The range of measured pump pulse energies are indicated in (**a**,**c**,**e**). The corresponding ASE threshold values as shown in (**b**) (QDs), (**d**) (QRs) and (**f**) (NPLs) are at the pump fluences of ≈542 *μ*J*cm*^−2^, ≈25 *μ*J*cm*^−2^ and ≈15.42 *μ*J*cm*^−2^, respectively.

Figure 2 represents the ASE indication with 2*ω* pumping. The arrows in each graph Fig. 2a (QDs), Fig. 2c (QRs) and Fig. 2e (NPLs) represent the energy range on the sample, considering the interaction length to be 1.5 mm. The nanoparticles showed strong broadband fluorescence for low pump energy and clear evidence of ASE at higher pump energies at peak wavelengths of 632 nm (QDs), 623 nm (QRs) and 572 nm (NPLs), respectively. ASE threshold energies and fluences were evaluated from Fig. 2b,d,f. In this case, the threshold values for all three films QDs, QRs and NPLs are as low as ≈0.285 *μ*J, ≈0.012 *μ*J and ≈0.009 *μ*J, respectively. Taking into account the length and the width of the illuminated area of the line focus on the sample, this corresponds to pump fluences of ≈542 *μ*J*cm*^−2^, ≈25 *μ*J*cm*^−2^ and ≈15.42 *μ*J*cm*^−2^, respectively. These values agree well with data given in previous literatures^17,24^. The threshold value for our NPLs is lower than previously reported 5 monolayer thick CdSe, which is 28 *μ*J*cm*^−2^ ^25^.

The particles of all dimensionality showed a slight variation in the emission wavelength during pump power variation. However, since this shift is within the resolution limit of the used spectrometer, this effect was not investigated further in the present paper. Consequently, for the evaluation of the gain curves only the maximum emission peaks were taken. The results are given in Fig. 2(b,d,f). The QDs and the NPLs show degradation at higher pump fluences. For the QDs generally, the necessary pump fluence is one to two orders of magnitude higher than for QRs and NPLs. In this case the degradation can be attributed to the damage threshold of the material layer. For the NPLs the absorbed energy is highest so that the damage threshold is drastically reduced. Degradation was observed here already for pump energies above ≈0.45 *μ*J (for QDs), ≈0.038 *μ*J (for QRs) and ≈0.0135 *μ*J (for NPLs). These correspond to the fluences values of 918 *μ*J*cm*^−2^, 59 *μ*J*cm*^−2^ and 20 *μ*J*cm*^−2^, respectively.

**Figure 3:**
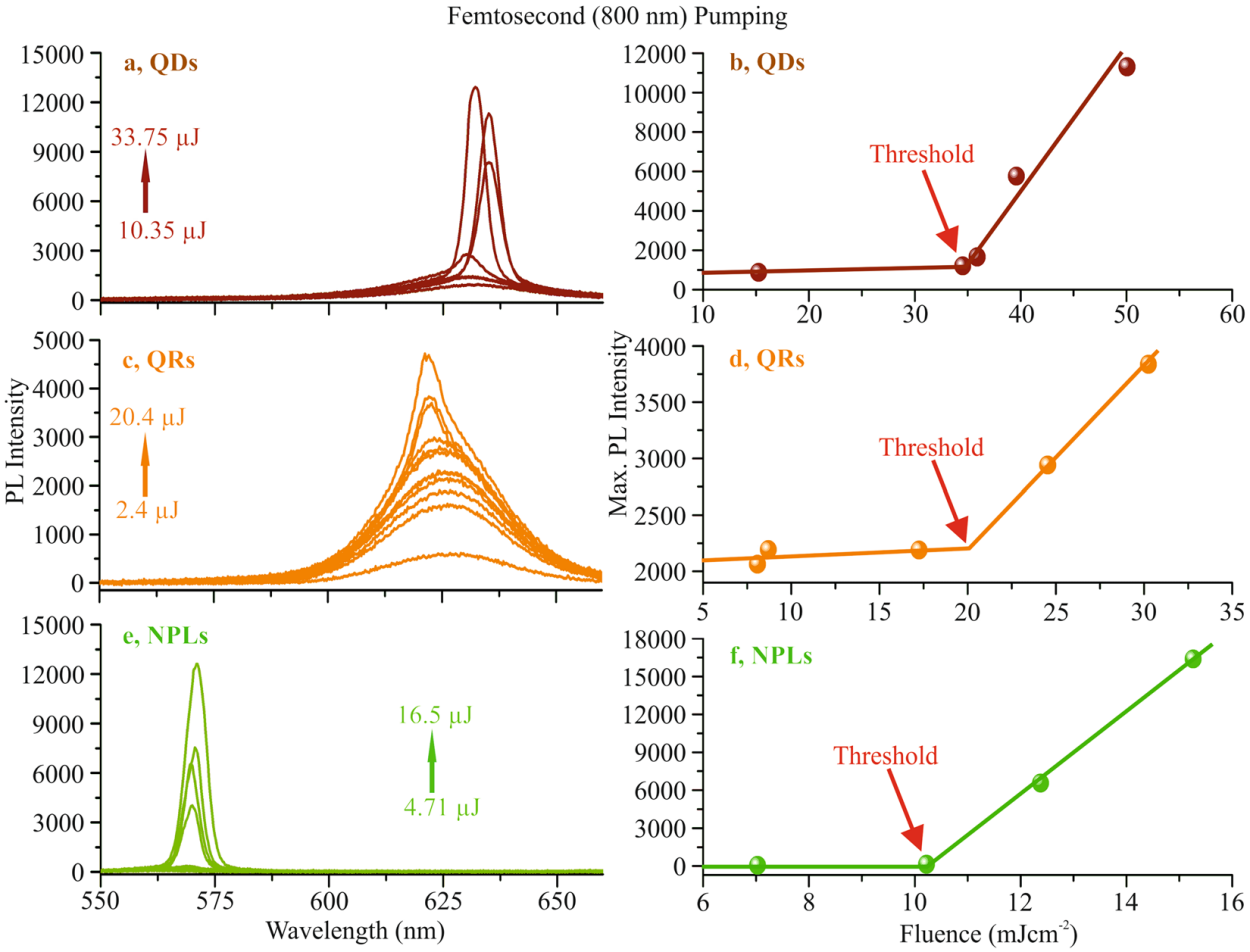
Femtosecond fundamental pumping emission of different nanoparticles. The energy variation with minimum and maximum in each nanoparticles coated film is shown by arrow in each graph (a for QDs, c for QRs and e for NPLs). The (**b**,**d**,**f**) represent the threshold values at the maximum peak intensity wavelength for each particle. The threshold values of QDs (**b**), QRs (**d**) and NPLs (**f**) are at pump fluences of ≈35 mJ*cm*^−2^, ≈20 mJ*cm*^−2^ and ≈10.5 mJ*cm*^−2^, respectively.

An additional study was carried out using femtosecond pumping at a wavelength of 800 nm. Due to the two-photon absorption required for pumping the threshold pump pulse energies are significantly higher than in the previous case. The energy range on the sample is again indicated by the arrows in each graph for the different nanoparticles. The lowest pump energy represents the energy at which clearly fluorescence could be detected. The energies were then increased until the films were bleached. The ASE threshold energy again was taken when clear onset of the narrow ASE peak occurred. Considering the interaction length of 1.5 mm, the threshold values of QDs, QRs and NPLs to be ≈22.5 *μ*J, ≈11.04 *μ*J and ≈6.75 *μ*J, respectively as shown in Fig. 3b,d,f. This corresponds to threshold pump fluences of ≈35 mJ*cm*^−2^, ≈20 mJ*cm*^−2^ and ≈10.5 mJ*cm*^−2^, respectively. These values are in line with data given in the literature^26^.

The above experiments, using femtosecond one-photon pumping at the second harmonic (2*ω*) and two-photon pumping at fundamental (*ω*) wavelengths of the titanium-sapphire, served for referencing the spectral measurements with common results from literature and to demonstrate that clear signals of ASE were obtained from all types of nanoparticles under investigation. In case of femtosecond pumping only energy/fluence variation measurements were carried out at room temperature. The main goal of this contribution, however, was the demonstration of ASE signals from the different nanoparticles, using nanosecond pumping at room temperature, and the demonstration of high intrinsic gain in QRs and NPLs material films due to their enhanced absorption properties as explained above.

**Figure 4:**
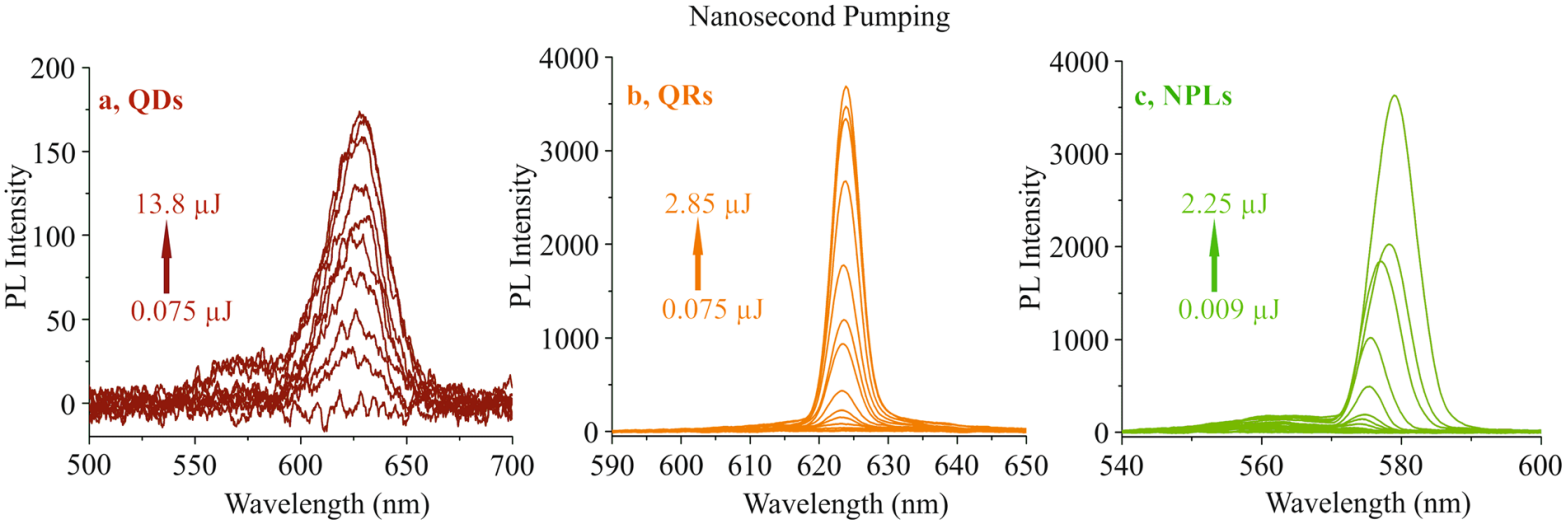
The comparison between nanosecond pumping of (**a**) QDs, (**b**) QRs and (**c**) NPLs. The arrows in each graph (red for QDs, orange for QRs and green for NPLs) represents the energy range, adopted for each case. The QRs and NPLs show clear indication of ASE at the peak wavelengths of 624 nm and 579 nm, respectively but only fluorescence is detected from QDs at the peak wavelength of 628 nm.

Under nanosecond pumping, all three spin-coated nanoparticles films were illuminated up to the damage threshold of the samples, as shown in Fig. 4a–c, but only the spectra of QRs and NPLs exhibit clear indications of ASE. Note the different wavelength scales in Fig. 4. The QDs only show a sign of fluorescence. Damage threshold of the sample, i.e degradation of emission spectra were observed for the fluences values of 4 mJ*cm*^−2^, 5.5 mJ*cm*^−2^ for QRs and NPLs, respectively. Obeying the Brus theory^10^, the spherical QDs with small diameter have a larger energy band gap in comparison to bigger ones. This also requires higher pulse energy for the excitons, increasing the timescale for the normal emission process. During this process, AR process can occur, disturbing the electron-hole relaxation mechanism. This effect is not observed in QRs and NPLs due to the fact that their 1-D and 2-D degrees of freedom let the emission process occur faster than AR. The ASE threshold value shown in Fig. 4a,b are ≈1.3 mJ*cm*^−2^ (a) and ≈1.54 mJ*cm*^−2^ for QRs and NPLs respectively. In contrast to femtosecond pumping, the ASE thresholds here appear to be very similar, despite the difference in size of the extended shell structures. It is assumed that due to the larger size of the NPLs, the pumping process is more efficient since the shell structure acts as an optical antennae for the pump radiation. This effect is corroborated by the experimental results of femtosecond pumping. However, for nanosecond pumping, the situation is different with respect to the pumping wavelength and of course the pulse duration. First, the absorption cross sections for QRs and NPLs may differ slightly which may result in different pump efficiencies. Second, and this is more decisive, the materials significantly accumulate heat during the nanosecond pulse duration of the pump pulses. The heat cannot be transferred within this time duration to the substrate. Due to their extended shell structure, the heating process is more pronounced for the NPLs than for the QRs^27^. This effect is visible in the experimental results by an increase of the ASE threshold of the NPLs. As a result, the ASE threshold of QRs and NPLs appear to be in the same order of magnitude, in contrast to the results of femtosecond pumping. The investigation of the influence of pump pulse duration for different pump laser wavelengths will subject to a more detailed study.

**Figure 5:**
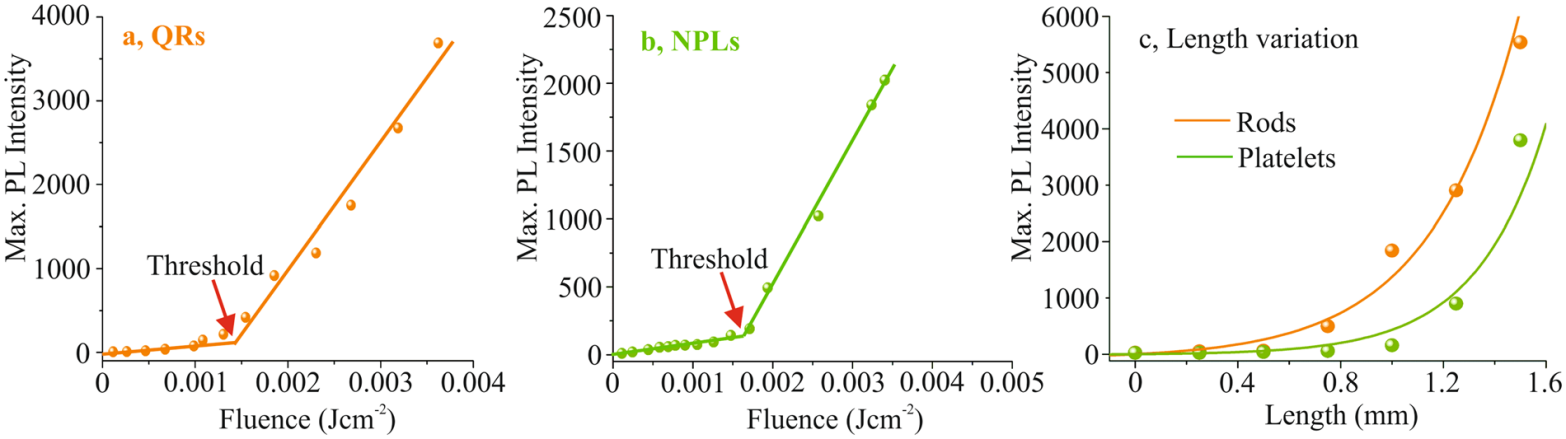
The QRs and NPLs show ASE thresholds at ≈1.3 mJ*cm*^−2^ (**a**) and ≈1.54 mJ*cm*^−2^ (**b**), respectively. (**c**) Represents the slit variation performance of QRs and NPLs corresponding to the pump pulse energy at the sample to be ≈2.25 *μ*J, with an interaction length of 1.5 mm. The QRs needs 0.5 mm opening of the slit for ASE whereas NPLs needs 0.85 mm.

Due to the absence of ASE in QDs with nanosecond pumping, the variation of the interaction length, i.e. the length variation of the pumped zone, was carried out only for QRs and NPLs. In this experiment the pump fluence was set to 3.3 mJ*cm*^−2^ for both, QRs and NPLs, in order to stay clearly below the damage thresholds of the samples. For the applied pulse energy clear ASE could be detected for the full interaction length. As demonstrated, both QRs (orange) and NPLs (green) have no emission until 0.5 mm of slit opening. The gain values of QRs and NPLs calculated by fitting the points in Fig. 5(c) are 29 *cm*^−1^ and 37 *cm*^−1^, respectively. These values are in the same order of magnitude of QD gain, measured under nanosecond pumping, but using a distributed Bragg reflector to benefit from enhanced modal gain^19^.

In summary, light amplification properties of CdSe/CdS QDs (emission wavelength 620 nm), QRs (emission wavelength 621 nm) and NPLs (emission wavelength 554 nm) at ambient temperature were investigated. The thin films (150 nm–190 nm) of all three nanoparticles were spin-coated on glass substrates. Three pumping regimes have been investigated: (i) femtosecond pumping using the second harmonic of a titanium-sapphire laser with 50 fs pulse duration at 400 nm, (ii) femtosecond pumping at the fundamental wavelength of the femtosecond laser at 800 nm, and (iii) nanosecond pumping by third harmonic of a q-switched Nd:YAG laser (355 nm) with 6 ns pulse duration. We have observed that ASE of all nanoparticles can be achieved at room temperature for femtosecond one-photon and two-photon pumping. The emission threshold for each nanoparticle sample was measured on the basis of pump fluence variations. In comparison, 2*ω* pumping produced the lowest threshold power for ASE in all three nanoparticles, which agrees well with values given in the literature. For nanosecond pumping, ASE at room temperature was demonstrated for QRs and NPLs due to their enhanced absorption properties, opening the way for practical applications of these materials. The length variation of the pumped zone on the sample in this case yielded gain coefficients of 29 *cm*^−1^ for QRs and 37 *cm*^−1^ for NPLs.

## Methods

### Synthesis

The CdSe/CdS nanoparticles, namely core/shell QDs, core/shell QRs and core/crown 5 monolayer NPLs are prepared by the procedure described in following journals^28–33^.

- CdSe/CdS core/shell QDs: These particles were synthesized following the procedure^30^, which consists of two step synthesis with preparing CdSe seeds, followed by coating with one CdS layer via successive ionic layer adsorption reaction (SILAR). After the synthesis and purifications the QDs were dispersed in 4 mL toluene.
- CdSe/CdS core/shell QRs: CdSe seeds were synthesized following the procedure of ^29^. Subsequently, CdS shell were prepared by a seeded-growth approach based on the reaction procedure as mentioned in the same literature. The mixture of 0.06 g of CdO (99.99%), 3.0 g of TOPO (99%), 0.28 g of ODPA (99%) and 0.08 g of HPA (99%) were placed in a three neck flask and degassed under vacuum for 1 h at 150 °C. Afterwards, the reaction temperature was raised to 300 °C under argon atmosphere. At the same temperature, 1.8 mL of TOP was injected and the solution was heated to 380 °C under argon. Upon achieving this temperature, a sulfur precursor solution (0.130 g S dissolved in 1.8 mL TOP) consisting of CdSe seeds in toluene (concentration of CdSe was 400 *μ*M) was quickly injected. The temperature was decreased down to 270–300 °C and the reaction was continued for 8 minutes before removing the heating mantle. The products were purified by centrifugation 3600 g (rcf) for 15 min and the precipitate was dispersed in 4 mL toluene.
- 5 ML CdSe/CdS core/crown NPLs: These particles were synthesized according to the procedure described by^28^ with little modifications. A mixture of 170 mg cadmium myristate *Cd*(*myr*)_2_ and 14 mL of ODE (90%) inside a 25 mL three neck flask was degassed under a vacuum at 100 °C for one hour with continuous stirring. Afterwards, the temperature of the reaction medium was increased up to 250 °C under argon flow, followed by injecting 1 mL of selenium precursor (12 mg Se dispersed in 1 mL of ODE) into the mixture. Exactly one minute after Se injection, 120 mg of *Cd*(*OAc*)_2_.2*H*_2_*O* was added to the mixture. After 10 min at 250 °C, the reaction was stopped and 1 mL oleic acid was added. The CdSe NPLs were separated by using 3:1 hexane:ethanol mixture. In a mean time, an anisotropic CdS growth mixture was prepared by dissolving 480 mg of *Cd*(*OAc*)_2_, 340 *μ*L of oleic acid and 2 mL of ODE at 150 °C for 15 min. 3 mL of a 0.1 M sulfur (in ODE) precursor solution was added into the mixture once the reaction temperature was lowered down to room temperature. The precipitate of CdSe NPLs was redispersed in 10 mL ODE and was placed inside a flask for the next step of CdS crown growth. After degassing at 100 °C for an hour, the mixture temperature was elevated to 240 °C and an anisotropic CdS growth mixture was continuously injected (using a syringe pump) at a rate of 8 mL/h into the above solution for 10 min. The heating mantle was removed. The core/crown nanoplatelets were separated from the mixture by adding 15 mL of ethanol followed by centrifugation at 3600 g (rcf) for 15 min. The precipitate was dispersed in 4 mL hexane.

**Figure 6:**
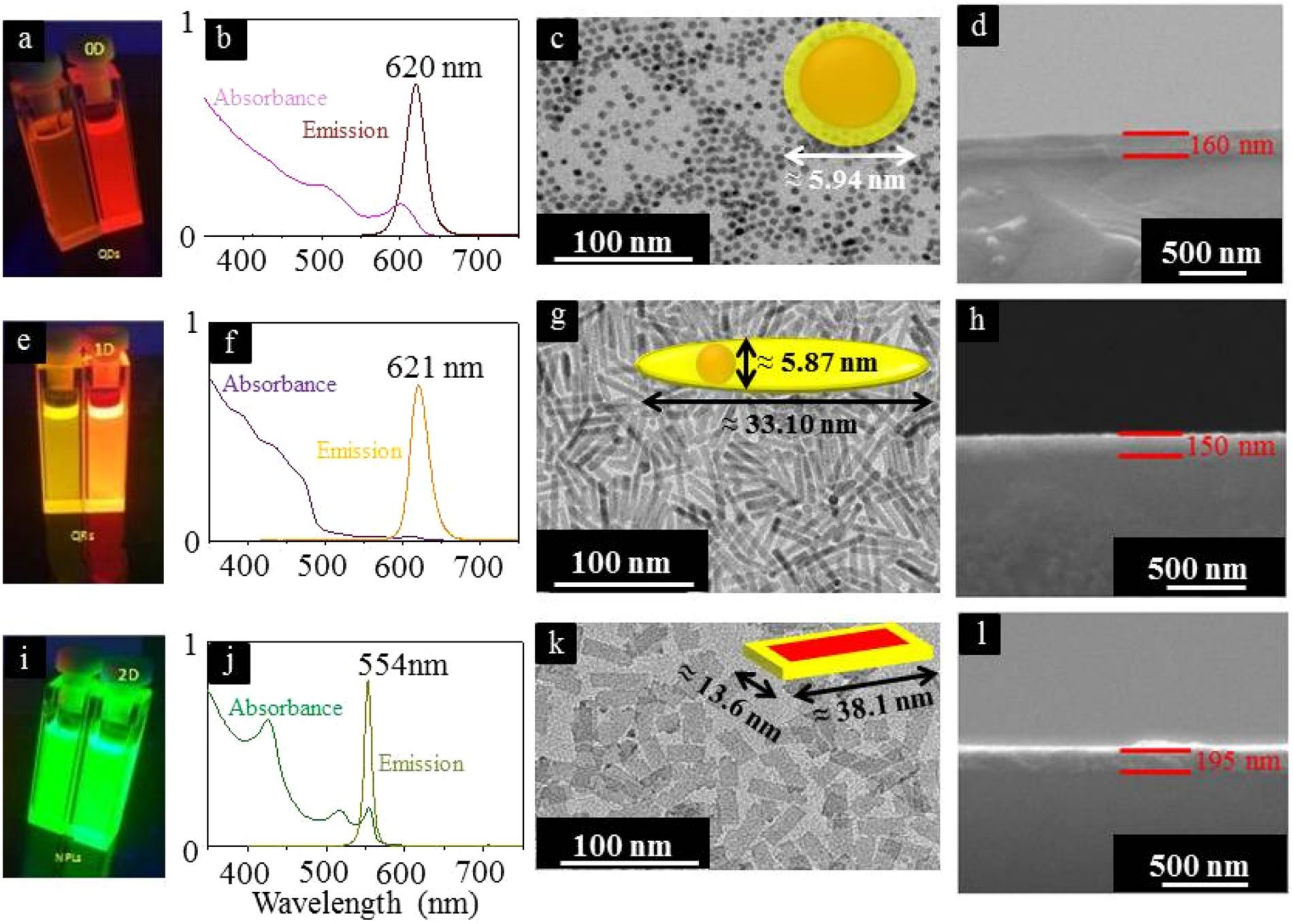
CdS core (left), CdSe/CdS (right) raw materials of core/shell QDs (**a**), core/shell QRs (**e**) and core/crown NPLs (**i**) respectively. The QDs has an absorption peak at 620 nm (**b**), QRs at 621 nm (**f**) and NPLs at 554 nm (**j**). The TEM image of each particles shows the QDs with a diameter of 5.94 nm ± 0.46 nm (**c**), the QRs, with a diameter of 5.87 nm ± 0.59 nm with length of 33.10 nm ± 2.84 nm (**g**) and the NPLs with a length of 38.1 nm ± 5.2 nm with width of 13.6 nm ± 2.1 nm (**k**). The NPLs have a thickness of only 5 monolayer (≈1.5 nm). In the schematic diagrams of QDs (**c**), QRs (**g**) and NPLs (**k**), the red structure represents CdSe core and yellow structure represents Cds shell/crown. The spin-coated films of QDs (**d**), QRs (**h**) and NPLs (**l**) are about 150–190 nm thick.

The prepared raw materials are shown in Fig. 6a,e,i (QDs, QRs and NPLs respectively).

### Absorption and emission

The absorption and emission spectra were measured using an Agilent Cary 5000 absorption spectrophotometer in transmission mode. The photoluminescence emission spectra of the samples were recorded using a Horiba Fluoromax-4 spectrometer in a 1 cm quartz cuvette using hexane, toluene (UV-vis spectroscopy grade). QDs has an absorption peak at 620 nm (Fig. 6b), QRs at 621 nm (Fig. 6f) and NPLs at 554 nm (Fig. 6j).

### Electron Microscope Imaging

TEM images were obtained using a FEI Tecnai G2 F20 TMP (Cs = 2 mm, CC = 2 mm) equipped with a 200 kV field-emission gun. The samples were purified 3–4 times via precipitation with methanol/ethanol and re-dispersion in toluene/hexane prior to TEM grid preparation. Subsequently, one drop of the purified sample was placed on a carbon coated copper TEM grid and dried under ambient conditions. The obtained TEM pictures are shown in Fig. 6c,g,k. The schematic diagrams in those figure as red and yellow represent CdSe (core) and CdS (shell/crown) respectively. The QDs have a diameter of 5.94 nm ± 0.46 nm, the QRs have diameter of 5.87 nm ± 0.59 nm with length of 33.10 nm ± 2.84 nm and the NPLs have length of 38.1 nm ± 5.2 nm with width of 13.6 nm ± 2.1 nm. The NPLs have a thickness of only 5 monolayer (≈1.5 nm).

### Film Deposition

A small glass slide (BAK coverslips) of 1 × 18 × 10.15 *mm*^3^ dimensions were cleaned thoroughly by sonicating with acetone and then deionised water for a minute. A total amount of 50 *μ*L QDs, QRs and NPLs nanoparticles were spin coated on the glass slides separately at a speed of 10 rps for 10 seconds. The film thickness of each material is shown in Fig. 6d (QDs), h(QRs) and l(NPLs). These films are about 150–195 nm thick. No air inclusions, voids or defects are visible in the films and according to an additional measurement the optical density of the films is the same, about 0.2. Therefore it is assumed that the packaging density is similar for all three materials (check supplementary notes for more information).

### ASE Measurements

A set up as shown in Fig. 1a consists of a laser source that was passed through a cylindrical lens (f = 5 cm) to the sample. A slit barrier was used for manually controlling the slit width. The laser focus spot with length of 10 mm and a width of 45 *μ*m, was focused at the edge of the film in such a way that when the slit was opened completely, only half of the length focus was on the film. An optical fibre was allocated perpendicularly, very close to the edge of the film to measure the PL intensity of the illuminated area. Figure 1b shows an experimental photo of excited QRs. The integration time of our spectra were 333 ms for nanosecond pumping and 1000 ms for femtosecond pumping.

- Nanosecond pumping: The films were pumped by a laser source of 355 nm with a pulse duration of 6 ns at a repetition rate of 14 Hz. Two different sets of experiments were investigated:

#### Power variation

The slit was kept completely open to let half of the laser beam to excite the sample. The ASE threshold energy and the fluorescence spectra were collected by changing the laser intensity to different levels.

#### Interaction length variation

Once the ASE was achieved, the power was kept constant at one particular value and the slit was opened slowly until the emission was detected. The fluorescence spectra was measured at every 500 *μ*m opening.

- Femtosecond pumping: The power variation experiment was repeated with 50 fs pumping at two different sets of experiments: (i) Fundamental (800 nm) at repetition rate of 50 Hz, (ii) 2*ω* (400 nm) at repetition rate of 20 Hz.

## Electronic supplementary material

Supplementary Notes
