# Supplementary material for: Low Threshold Room Temperature Amplified Spontaneous Emission in 0D, 1D and 2D Quantum Confined Systems
Source: Sci Rep. 2018 Mar 2;8:3962. doi: 10.1038/s41598-018-22287-9 (PMC5834608; doi:10.1038/s41598-018-22287-9)
Supplement: Supplementary file 1 — Supplementary Notes [file 41598_2018_22287_MOESM1_ESM.pdf]

Supplementary Notes

Title: Low Threshold Room Temperature Amplified Spontaneous Emission in 0D, 1D and 2D Quantum Confined Systems

Authors: Parva Chhantyal, Suraj Naskar, Tobias Birr, Tim Fischer, Franziska Lübkemann, Boris N. Chichkov, Dirk Dorfs, Nadja C. Bigall and Carsten Reinhardt

In our experiments, solid films of particles of the CdSe/CdS material system have been used, exhibiting thicknesses between 150 nm and 190 nm. The high packaging densities are required to compensate for Auger losses. Stimulated gain in QDs is limited by intrinsic Auger recombination, which appears whenever two or more electron-hole pairs are present in one QD. On the other hand, stimulated emission or lasing from a QD requires at least two electron-hole pairs, and therefore the Auger losses are intrinsically unavoidable. However, lasing can still be achieved if cumulative stimulated processes among many QDs occur faster than the Auger recombination. Klimov et al. have shown that the rate of the stimulated emission buildup is proportional to the dot concentration in the sample[3]. It has been demonstrated that by close packaging of QDs into solid state films, it is possible to obtain QDs concentrations that are sufficiently high for the optical gain to successfully compete with the Auger decay.

The films used in the present experiment have been fabricated by spin-coating. Before coating of the films, the quantum materials are suspended in organic solution, stabilized by organic agents. Optical density (OD) has been measured on both, liquid solution and spin-coated films. Measurements refer to a wavelength of 400 nm. The solutions have the same values of OD according to the measurements with the Agilent Cary 5000 absorption spectrophotometer. For the films, we found values of 0.181 (QDs), 0.204 (QRs), and 0.197 (NPLs). These data support information that allows concluding that the densities of the films are of the same order as it should be expected for a closely packed material film of CdSe/CdS.

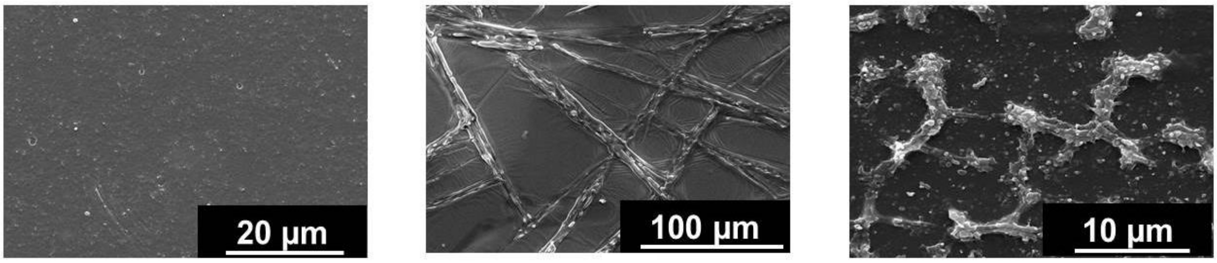

a b c

Supplementary Figure S 1: Top view of the spin-coated films of QDs (a), QRs (b) and NPLs (c). The CdSe/CdS material forms closed films of 150–190 nm thickness directly on the glass slides. The aggregated structures are the residues of stabilizing agents of lower optical and material density that deposited onto the actual CdSe/CdS film during the spin-coating process. In contrast, aggregation of the CdSe/CdS dots, rods, and platelets results in the closed, closely packed, and homogeneous CdSe/CdS film.

However, as can be seen from Fig. S1 the solid quantum films are covered by residues of the organic stabilizers. The visible aggregation is that of these unavoidable residues of organic material of the necessary stabilizer agents, which are used in the chemical synthesis. However, due the high laser pump intensities the structures of stabilizing agents are evaporated during the first laser pulses. The dot, rod and plate shaped particles with sizes in the range of a few (5.94 nm) to a few tens (maximum length of a platelet is 38.1 nm) are not visible in these images. The particle films under consideration in this contribution form a closed film underneath the stabilizer structures. This can be seen in the fluorescence image Fig. S2, showing a spin-coated film of CdSe/CdS rods under UV illumination.

Fig. S2 further shows the occurrence of edge beats during the spin-coating process. Since we measure the light emission from the sample edges, this edge quality is of importance for the experiment. Nevertheless, it is easily possible to position the laser line focus to a position where the edge beats allow sufficient out-coupling of light for reliable measurements.

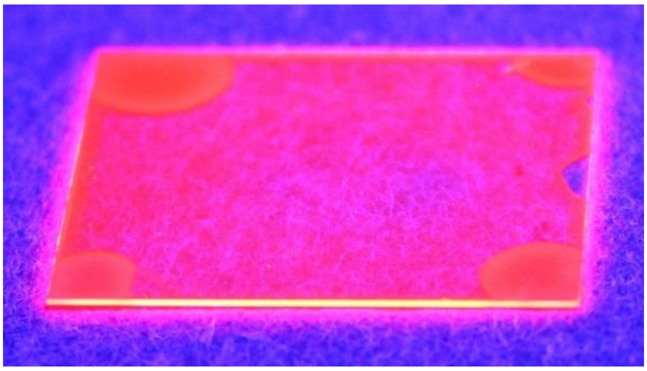

Supplementary Figure S 2: Top view of the spin-coated films of QRs. The side length of the square glass substrate is 18 mm. The CdSe/CdS film of 150 nm thickness can be identified by homogeneous fluorescence under illumination with a wavelength of 365 nm. The aggregated stabilizer structures are visible as a granular structure on top of the films which evaporates during the first laser pulses. Visible are also the edge beats which occur during the spin-coating process.
